# A clinical profile of pediatric patients admitted for thryotoxicosis in a tertiary hospital using the 1992 Burch and Wartofsky criteria

**DOI:** 10.1186/1687-9856-2013-S1-P152

**Published:** 2013-10-03

**Authors:** Josephine Leiya Marie F Salud, Sylvia C Estrada

**Affiliations:** 1 University of the Philippines-Philippine General Hospital, Department of Pediatrics, Manila, Philippines

## 

Thyrotoxicosis is the clinical syndrome of hypermetabolism that occurs with increased levels of thyroid hormones. Thyroid storm (TS) represents the extreme manifestation of thyrotoxicosis. Medical charts of all pediatric patients with the diagnosis of “Thyroid Storm or “Impending Thyroid Storm” (ITS) from 2004 to 2010 were retrieved. Patients with a BWC of 25 and above were included. Charts with incomplete data were excluded. Fourteen patients (13 females and 1 male) between 12-18 years of age were included. All had goiter and were biochemically hyperthyroid. The most common chief complaint was dyspnea(57%). The most common precipitating factors were infection and noncompliance to medicines(43%). The mean length of hospital stay was 6.2±4 days. The most common organ system dysfunctions were diarrhea(50%), tachycardia(43%) and hyperthermia(38%). Eight(57%) fit the criteria for TS and 6(43%) for ITS. The mortality rate was 29%.

The hyperthyroid pediatric patients were admitted due to organ system decompensation secondary to thyrotoxicosis and/or concomitant disease. The levels of thyroid hormone did not distinguish between thyrotoxicosis and TS nor predict the outcome. The most common intercurrent diseases were pneumonia and acquired cardiac disease, the symptoms (fever, tachypnea, tachycardia) of which overlapped with symptoms of the hyperthyroid state. The clinical changes in cardiovascular hemodynamics that occur in patients in thyrotoxicosis aggravates cardiovascular disease, increasing mortality risk. The patients who were admitted with heart failure signs eventually expired.

Wartofsky emphasizes that the diagnosis of ITS and TS is clinical and based on the severity of the symptoms and signs of thyrotoxicosis and presence of functional decompensation of organ systems. The potential high mortality necessitates early treatment. The mortality rate in our study is 29%(4/14 cases), which is higher than that reported among adults(11.3% of 71 cases) in the same tertiary hospital but within the range of 20-30% in the adult population in first world countries. Only two(50%) of those who died had high BWC scores(range 70-85) questioning now the usefulness of BWC as a helpful tool in assessing disease severity among adolescents.

In conclusion, the clinical profile of pediatric patients in thyrotoxicosis using the BWC was reviewed. A validity study on the use and applicability of BWC for pediatric patients is recommended.

